# Self-catalyzed Growth of InAs Nanowires on InP Substrate

**DOI:** 10.1186/s11671-017-1825-2

**Published:** 2017-01-13

**Authors:** Bang Li, Xin Yan, Xia Zhang, Xiaomin Ren

**Affiliations:** State Key Laboratory of Information Photonics and Optical Communications, Beijing University of Posts and Telecommunications, Beijing, 100876 China

**Keywords:** Nanowires, Vapor–liquid–solid (VLS), Kink, Self-catalyzed

## Abstract

We report on the self-catalyzed growth of InAs nanowires on InP substrate by metal-organic chemical vapor deposition. At a moderate V/III ratio, tapered nanowires are obtained, suggesting a strong surface diffusion effect. Dense twin faults are observed perpendicular to the nanowire growth direction due to the fluctuation of In atoms in the droplet originating from the surface diffusion effect. At a lower V/III ratio, the nanowires exhibit kinking, which is associated with a high adhesion due to a large sticking coefficient of TMIn. The twin faults are dramatically suppressed and even completely eliminated in the NW branch after kinking, which is attributed to a stable In supply with a negligible diffusion effect. This work provides a method for the fabrication of defect-free InAs nanowires.

## Background

Recently, III/V nanowires (NWs) have attracted increasing attention for their potential applications in future devices [1–7]. High-performance NW devices require high quality NWs with controlled morphology and perfect crystal phase. For example, NW solar cells with uniform diameter achieve high conversion efficiency [8]. High-speed field effect transistors require twin-faults-free NWs avoiding from electron trapping [9]. InAs NWs are particularly promising for high-performance electronic and optoelectronic devices due to the high electron mobility and broad spectra response. However, the control of the morphology and crystal phase of InAs NWs still remains a challenge. Particularly, stacking faults and twin defects are commonly observed in InAs NWs, which dramatically limit their applications in high-performance devices [10, 11]. Up to date, defect-free III-V NWs have been mainly obtained by controlling the growth conditions, varying the catalyst size, as well as changing the substrate orientation [12–14]. Besides, single-crystalline crystal structure has also been obtained in tilted or kinked NWs. For example, tilted Ge, ZnSe, and GaAs NWs have been reported to be SFs-free [15–17]. Pure zincblende (ZB) phase has been obtained in GaAs, InP, and Ge NWs after kinking [18–21]. The tilted or kinked NWs typically have a low-index orientation, which favors pure ZB phase. However, the research on tilted or kinked InAs NWs is still limited. Moreover, most of the reported InAs NWs are grown via Au-catalyzed method, which may lead to an unintentional Au contamination and degrade the device performance. In this paper, we demonstrate the growth of InAs NWs on InP substrate by self-catalyzed growth method. NWs are grown under different temperature and V/III ratio. Twin-free NWs are obtained via a spontaneous kinking, and the related mechanism is discussed.

## Methods

The growth was carried out in a Thomas Swan close-coupled showerhead-metal-organic chemical vapor deposition (CCS-MOCVD) reactor at the pressure of 100 Torr. Before NW growth, an InP (111)B substrate was placed in the reactor and annealed in situ at 645 °C in phosphine ambient for desorption of surface contaminants. Trimethylindium (TMIn) and arsine (AsH_3_) were used as precursors. Hydrogen served as the carrier gas. In droplets were formed by switching off PH_3_ and depositing TMIn for 45 s at 390 °C. Then, TMIn precursor was switched off for 10 s of soak time. InAs NWs growth began when TMIn and AH_3_ were introduced again into the reaction chamber simultaneously. The input V/III ratio was adjusted by varying the AsH_3_ flow while keeping the TMIn flow constant at 32 μmol/min. After growth, the samples were cooled down in H_2_ ambient. Four samples were grown in the experiment. Samples A, B, and C were grown for 900 s at a V/III ratio of 35 and growth temperature of 420, 400, and 380 °C, respectively. Sample D was grown for 900 s at a V/III ratio of 18 and growth temperature of 380 °C.

The morphological and structural characteristics of the samples were characterized by scanning electron microscopy (SEM) and transmission electron microscopy (TEM). Individual NWs for TEM observations were prepared by ultrasonicating the samples in ethanol for 5 min, followed by spreading drops from the suspension onto a holey carbon/Cu grid.

## Results and Discussion

**Fig. 1:**
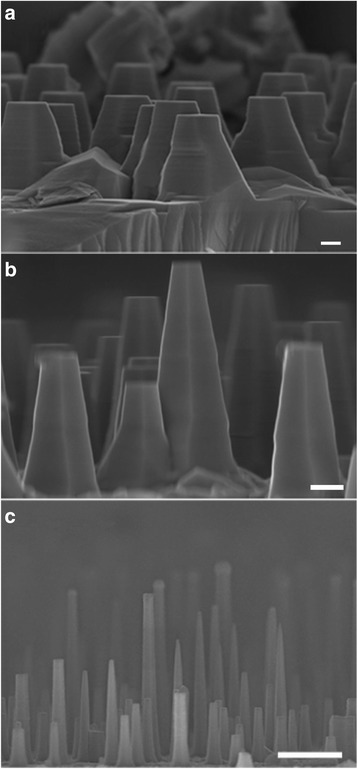
Cross-sectional SEM images of the InAs NWs from sample A, B, and C. The scale bars in (**a**–**c**) are 100, 200, and 1 μm, respectively

Figure 1a–c shows the cross-sectional SEM images of samples A, B, and C, respectively. All the NWs are vertical to the substrate, suggesting the <111> growth direction. Tapering is observed in all the three samples, indicative of the adatom diffusion during the NW growth. In comparison with sample C, samples A and B grown at higher temperature are more tapered, which is attributed to an enhanced diffusion of In adatoms from the substrate to the droplet [22]. The average length of NWs for samples A, B, and C is 0.63, 1.26, and 2.62 μm, corresponding to a growth rate of 0.7, 1.4, and 2.9 nm/s, respectively. The decreased growth rate with the increasing temperature is contrary to the traditional vapor–liquid–solid (VLS) growth that the growth rate usually increases with the increasing temperature due to the thermally activated behavior. This can be attributed to a decreased supply of In adatoms from the substrate due to an enhanced competition. The average diameter of the NW bottom for samples A, B, and C is measured to be 645, 501, and 270 nm, respectively, suggesting an enhanced radial growth as the temperature increases. As the NWs become thicker, the spacing between NWs becomes smaller. The competition of neighboring NWs for the In species on the substrate is enhanced due to an decreased collection area, resulting in a slower growth rate due to a decreased supply of In adatoms from the substrate [23].

**Fig. 2:**
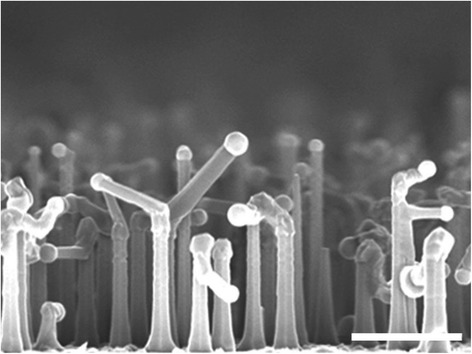
Cross-sectional SEM images of the InAs NWs from sample D. The *scale bar* is 1 μm

Figure 2 shows the cross-sectional SEM image of sample D. Different from the straight NWs in samples A, B, and C, kink is observed in some of the NWs, resulting in a change of growth direction. The kink phenomenon is attributed to a reduced V/III ratio. In the self-catalyzed InAs NW growth, the In droplet acts as the catalyst. With decreasing the V/III ratio, the sticking coefficient of TMIn increases and more In sticks to the surface. A high In content is expected to lower the liquid–vapor surface tension and thereby increase the work of adhesion. When the work of adhesion is high enough, a small perturbation (e.g., fluctuation of temperature or gas flow) is sufficient to change the form of the NW tip. In addition, the low surface energy (111) sidewalls are favorable for the droplet to wet. Under these combined conditions, when the faceted structure forms with a shrinking growth surface, the liquid droplet is easy to unpin, moving onto a sidewall (111) facet, and thus to continue to grow, forming a kink [24].

**Fig. 3:**
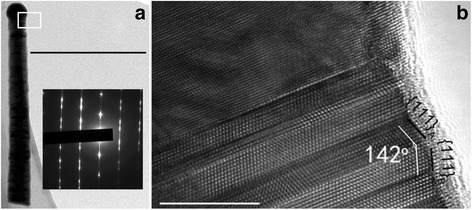
**a** Low magnification TEM image of a single NW from sample C. The *inset* shows the SAED of the NW taken along the <110> zone axis. **b** HRTEM image of the NW with twinning defects. The scale bars in **a** and **b** are 500 and 10 nm, respectively

Figure 3 shows the TEM image of a single NW from sample C. A large number of twin faults are observed from the selective area electron diffraction (SAED), which have been widely reported in self-catalyzed InAs NWs [25, 26]. The twin faults usually occur with a transition of external facets during the nucleation formation and the free energy of the nucleus formation depends on the contact angle of the liquid droplet with respect to the solid–vapor facet [27]. Thus, the orientation of each critical nucleus determines whether a normal or a twin plane will form. As the energy difference between the normal and twin nucleus is small, small energy fluctuations during growth could give rise to randomly distributed twin planes. In this case, the sidewall facet perpendicular to the growth direction is {112}. {112} planes have a relatively high surface energy and can be considered as two {111} layers followed by a “correcting” step. The angle between the surfaces parallel to the NW growth direction is 38° (as shown in Fig. 3b), corresponding to {111}_A_ and {111}_B_ surfaces [28]. (The subscript “A” and “B” refers to “In” and “As” terminated surfaces, respectively.)

**Fig. 4:**
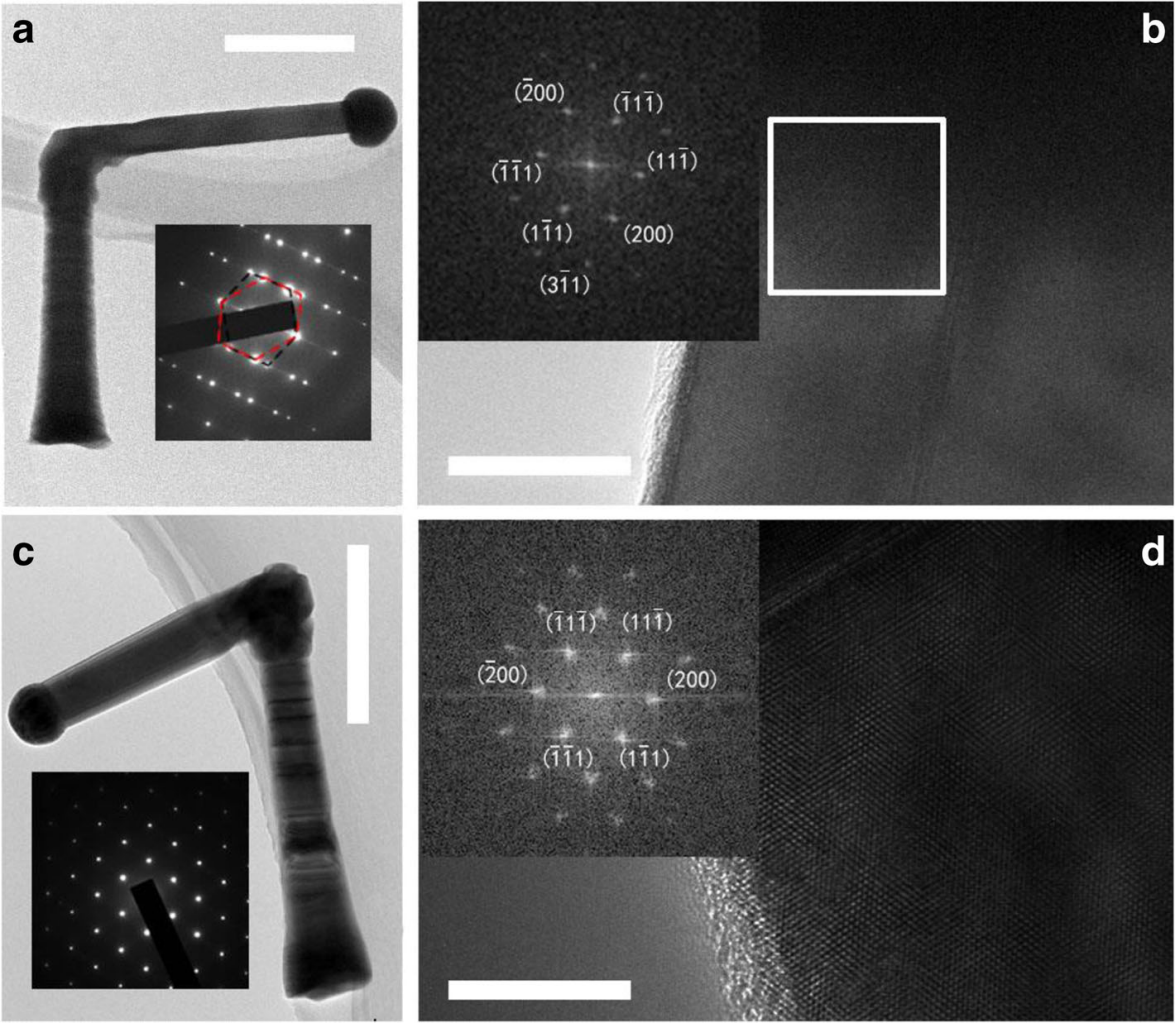
**a** Low magnification TEM image of a single NW from sample D. The *inset* shows the SAED of the NW after kinking taken along the <110> zone axis. **b** HRTEM image of the NW. The *inset* shows the FFT image of the *white marked area*. The <112> growth direction can be observed in the FFT image. **c** Low magnification TEM image of another single NW from sample D. The *inset* shows the SAED of the NW after kinking taken along the <110> zone axis. **d** HRTEM image of the NW. The *inset* shows the FFT image of the *white marked area*. The <111> growth direction can be observed in the FFT image. The *scale bars* in (**a**–**d**) are 500, 20, 500, and 10 nm, respectively

Figure 4 shows the TEM images of two kinds of NWs from sample D. Figure 4a shows a NW with an angle of 90° between the root and branch. The orientation of the branch is determined to be <112> according to the SAED. Figure 4b shows the high-resolution TEM (HRTEM) image between the droplet and NW. The branch exhibits zincblende (ZB) crystal structure despite several twins parallel with the growth direction of the branch. The twin formation probably occurs during the NW nucleation. As the NW continues to grow, the twins extend down the length of the NW. The twins form during the nucleation when the NW grows in the <111> direction and provide preferential addition sites that subsequently maintain the NW growth in the {112} direction. Compared with the twin faults perpendicular to the growth direction, the twin faults along the growth direction has smaller carrier scattering which has tiny influence on the mobility of NWs.

Figures 4c, d shows another kind of NWs from sample D. The growth direction is determined to be <111> according to the SAED. The branch after kinking exhibits defect-free ZB crystal structure without twins. The kink angle ranges from 70° to 137°, which has no relationship with the crystal orientation. This means that the kink is originating from other perturbation such as the In concentration fluctuation due to the surface diffusion rather than the stacking twins during the nucleation. It should be noted that the growth orientation of the branch will not change when the growth time is further prolonged.

It has been reported that the twin defects typically occurs with a transition of the external facets, and the transition takes place under the influence of the concentration in the droplet. The As species reach the growth point only by direct impingement, but the In species mainly come from direct impingement and surface diffusion from the substrate. In our experiment, as the growth parameters remain unchanged during the NW growth, the In species in the droplet from direct impingement is constant, but the In species collected from the substrate decrease with the NW height. Thus, the fluctuation of In species from the substrate contributes to the twin faults in the <111> root. When the NW grows to a certain height, kink occurs due to a small perturbation of growth conditions. Although the perturbation remains unclear, a sudden change of In concentration in the droplet may play an important role in the kink. That is, In diffusion from the substrate suddenly decreases, resulting in a sharp decrease of In atoms in the droplet. After kinking, the diffusion from the substrate becomes negligible and the direct impingement dominates. The stable supply of In species contributes to the pure crystal structure of the NW after kinking. The explanation can be supported by the TEM images in Fig. 4a, c, in which the NW roots are obviously tapered while the branch after kinking are uniform in diameter, suggesting that the diffusion effect is negligible after kinking.

## Conclusions

In conclusion, we demonstrate the self-catalyzed growth of InAs NWs on InP substrate by MOCVD. At a low V/III ratio, the NWs exhibit kinking, which is attributed to a high adhesion due to a large sticking coefficient of TMIn. The twin faults are dramatically suppressed and even completely eliminated in the NW branch after kinking, which is attributed to a stable In supply with a negligible diffusion effect. The twin-free InAs NWs are promising for high-performance electronic and photonic devices.

## Abbreviations

AsH_3_: Arsine
NW: Nanowire
SAED: Selective area electron diffraction
SEM: Scanning electron microscopy
TEM: Transmission electron microscopy
TMIn: Trimethylindium
VLS: Vapor–liquid–solid
